# Principles of Surgery

**Published:** 1896-06

**Authors:** 


**Principles of Surgery.** By N. Senn, M.D. Second Edition. Pp. xvi., 656. Philadelphia: The F. A. Davis Company. London: F. J. Rebman. 1895.

As five years have elapsed since the publication of the first edition of this work, the author has naturally found it necessary to add much new material, thus enlarging considerably the size and scope of the work; but the same originality and power have been displayed by Dr. Senn in the new parts of the book as in the old, and we can as warmly recommend the new edition as we did the former. More than fifty new illustrations have been added, and among them are five beautifully coloured plates showing stained bacilli. The *technique* of a number of operations is described and illustrated "for the special purpose of demonstrating, from a practical standpoint, the value of a thorough knowledge of the complicated reparative processes in the treatment of injuries and disease by surgical intervention."

The work is a model of what such works should be. It is written in a truly scientific spirit. Theory is maintained by experiment and illustration. The chapters on surgical tuberculosis should be read and studied by all surgeons and surgical students, for they teem with observations based on experiment and investigation, and are full of instruction and value. We know of no other book on the principles of surgery to compare with this one, which so worthily mantains the reputation of American authorship and publication.

